# Copy Number Analysis of 24 Oncogenes: *MDM4* Identified as a Putative Marker for Low Recurrence Risk in Non Muscle Invasive Bladder Cancer

**DOI:** 10.3390/ijms150712458

**Published:** 2014-07-14

**Authors:** Samanta Salvi, Daniele Calistri, Giorgia Gurioli, Elisa Carretta, Luigi Serra, Roberta Gunelli, Wainer Zoli, Valentina Casadio

**Affiliations:** 1 Biosciences Laboratory, Istituto Scientifico Romagnolo per lo Studio e la Cura dei Tumori (IRST) IRCCS, Meldola 47014, Italy; E-Mails: samanta.salvi@irst.emr.it (S.S.); daniele.calistri@irst.emr.it (D.C.); giorgia.gurioli@irst.emr.it (G.G.); valentina.casadio@irst.emr.it (V.C.); 2 Unit of Biostatistics and Clinical Trials, Istituto Scientifico Romagnolo per lo Studio e la Cura dei Tumori (IRST) IRCCS, Meldola 47014, Italy; E-Mail: elisa.carretta@irst.emr.it; 3 Pathology Unit, Morgagni Pierantoni Hospital, Forlì 47121, Italy; E-Mail: l.serra@ausl.fo.it; 4 Department of Urology, Morgagni Pierantoni Hospital, Forli 47121, Italy; E-Mail: fiorifo@tin.it

**Keywords:** NMIBC, recurrence, multiplex ligation probe amplification (MLPA), *MDM4*

## Abstract

Patients with non-muscle invasive bladder cancer (NMIBC) generally have a high risk of relapsing locally after primary tumor resection. The search for new predictive markers of local recurrence thus represents an important goal for the management of this disease. We studied the copy number variations (CNVs) of 24 oncogenes (*MDM4*, *MYCN*, *ALK*, *PDGFRA*, *KIT*, *KDR*, *DHFR*, *EGFR*, *MET*, *SMO*, *FGFR1*, *MYC*, *ABL1*, *RET*, *CCND1*, *CCND2*, *CDK4*, *MDM2*, *AURKB*, *ERBB2*, *TOP2A*, *AURKA*, *AR* and *BRAF*) using multiplex ligation probe amplification technique to verify their role as predictive markers of recurrence. Formalin-fixed paraffin-embedded tissue samples from 43 patients who underwent transurethral resection of the bladder (TURB) were used; 23 patients had relapsed and 20 were disease-free after 5 years. Amplification frequencies were analyzed for all genes and *MDM4* was the only gene that showed significantly higher amplification in non recurrent patients than in recurrent ones (0.65 *vs.* 0.3; Fisher’s test *p* = 0.023). Recurrence-free survival analysis confirmed the predictive role of *MDM4* (log-rank test *p* = 0.041). Our preliminary results indicate a putative role for the *MDM4* gene in predicting local recurrence of bladder cancer. Confirmation of this hypothesis is needed in a larger cohort of NMIBC patients.

## 1. Introduction

Bladder cancer is the 11th most common tumor worldwide [1]. The majority of cases present as non muscle invasive bladder cancers (NMIBC), which have a good long-term prognosis and low risk of progression and death. However, after initial transurethral bladder resection (TURB), about 80% of patients with NMIBC experience local recurrence within 5 years. The standard follow up of NMIBC involves a high number of cystoscopies, with consequently high healthcare costs and, often, low patient compliance [2].

Multiplicity, tumor size and prior relapse rate are the only recurrence-related parameters currently available for monitoring patients with bladder cancer [2]. However, this information would not seem to be accurate enough to ensure an adequate follow-up of individuals with stage Ta-T1 disease. There are four main mechanisms by which recurrence may occur: incomplete resection, tumor cell re-implantation, growth of microscopic tumors and new tumor formation [3]. The first two mechanisms are surgery related and can be avoided, while the growth of microscopic tumors or new tumor formation may be due to specific molecular alterations in urothelial cells. It would thus be useful to understand whether these molecular changes can be used to predict local bladder cancer recurrence.

Urothelial cancer is a heterogeneous disease and studies on epigenetics, gene expression and genomic profiles have brought to light several molecular subtypes with different clinical behavior [4,5,6,7,8]. On the basis of these findings, it can be hypothesized that the study of chromosomal instability in terms of gene copy number variations could help to identify subtypes of patients with different risk of progression and local recurrence [6,9].

In the present study we focused our attention on copy number changes in a panel of 24 oncogenes (*MDM4*, *MYCN*, *ALK*, *PDGFRA*, *KIT*, *KDR*, *DHFR*, *EGFR*, *MET*, *SMO*, *FGFR1*, *MYC*, *ABL1*, *RET*, *CCND1*, *CCND2*, *CDK4*, *MDM2*, *AURKB*, *ERBB2*, *TOP2A*, *AURKA*, *AR* and *BRAF*) known to be amplified in solid tumors and detected in DNA extracted from paraffin-embedded tissue of non muscle invasive bladder cancer. Our first aim was to identify some of these genes as recurrent predictive markers. Although several of these oncogenes, e.g., *MDM2*, *AURKA*, *MYC*, *HER2*, *CCND1* and *MDM4*, are known to be frequently amplified in bladder tumors of different grade and stage [10,11,12,13,14], their role as predictive markers in NMIBC has yet to be demonstrated. We chose the highly sensitive multiplex ligation probe amplification (MLPA) technique as it requires a low amount of DNA and is capable of analyzing several genes simultaneously [15].

## 2. Results and Discussion

### 2.1. Results

MLPA analysis was feasible for all 43 patients. The *AR* gene was excluded from the analysis because it was not evaluable in paraffin-embedded tissues. Gain frequencies in the overall case series varied from 0.46 for *MDM4* to 0.02 for *KDR*. Eight genes were not amplified (Table 1).

**Table 1:** Gain frequencies of each gene in the overall case series and in non recurrent and recurrent tumors.

Gene	Overall Series (43 Cases)	Non Recurrent Tumors (20 Cases)	Recurrent Tumors (23 Cases)	*p* Value *
*MDM4*	20/43	13/20	7/23	
	0.46	0.65	0.3	0.023
*MET*	15/43	10/20	5/23	
	0.35	0.5	0.22	0.052
*CDK4*	12/43	7/20	5/23	NS
	0.28	0.35	0.22	
*MYC*	12/43	8/20	4/23	NS
	0.28	0.4	0.17	
*TOP2A*	12/43	8/20	4/23	NS
	0.27	0.4	0.17	
*AURKA*	11/43	5/20	6/23	NS
	0.26	0.25	0.26	
*ERBB2*	9/43	2/20	7/23	NS
	0.21	0.10	0.30	
*MDM2*	8/43	6/20	2/23	NS
	0.19	0.3	0.09	
*CCND1*	6/43	4/20	2/23	NS
	0.14	0.2	0.09	
*BRAF*	6/43	3/20	3/23	NS
	0.14	0.15	0.13	
*DHFR*	5/43	3/20	2/23	NS
	0.12	0.15	0.09	
*FGFR*	4/43	2/20	2/23	NS
	0.09	0.01	0.09	
*CCND2*	4/43	1/20	3/23	NS
	0.09	0.05	0.13	
*KIT*	3/43	2/20	1/23	NS
	0.07	0.1	0.04	
*KDR*	1/43	0/20	1/23	NS
	0.02	0	0.04	
*MYCN*	0/43	0/20	0/23	NS
	0	0	0	
*ALK*	0/43	0/20	0/23	NS
	0	0	0	
*PDGFR*	0/43	0/20	0/23	NS
	0	0	0	
*EGFR*	0/40	0/20	0/23	NS
	0	0	0	
*ABL*	0/43	0/20	0/23	NS
	0	0	0	
*RET*	0/43	0/20	0/23	NS
	0	0	0	
*AURKB*	0/43	0/20	0/23	NS
	0	0	0	
*SMO*	0/43	0/20	0/23	NS
	0	0	0	

* Difference in gain frequencies between non recurrent and recurrent tumors (Fisher’s exact test); NS, not significant.

**Figure 1:**
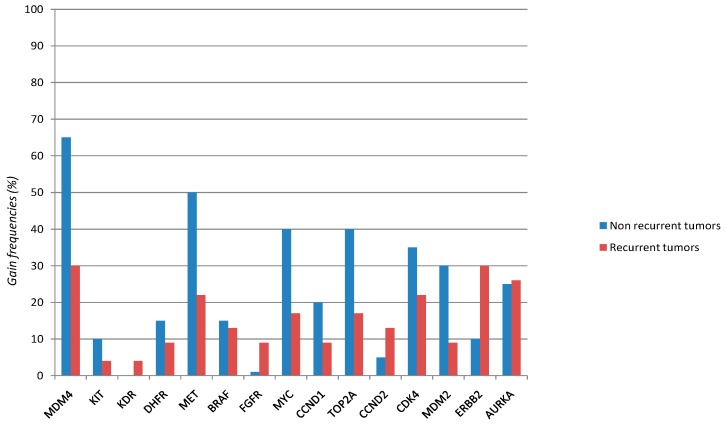
Gain frequencies in non recurrent *vs.* recurrent tumors.

A significant difference in the gain frequencies between non recurrent and recurrent tumors was observed for *MDM4* (0.65 *vs.* 0.3; *p* = 0.023) (Table 1; Figure 1). No statistically significant association was observed between the copy number variations of all 23 genes analyzed and stage or grade. However, results for *CCND1* were suggestive of a correlation with stage (*p* = 0.057, data not shown).

**Figure 2:**
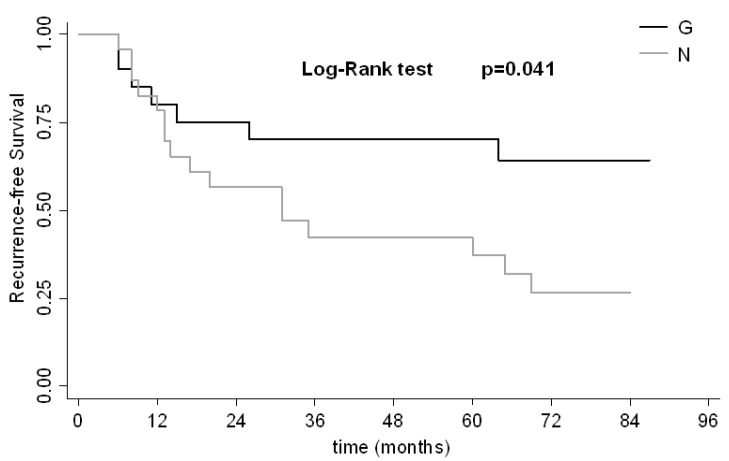
Recurrence-Free Survival (RFS)-Kaplan–Meier curves for *MDM4.* G, gain; N, normal.

A median recurrence-free survival (RFS) of 64 months (95% CI, 20-NR) was observed for the overall case series, with a median follow up of 86 months. Kaplan–Meier analysis of RFS was performed considering the copy number variations of *MDM4*. Five-year RFS was 70% (95% CI, 45.1–82.3) in patients carrying amplified *MDM4* compared to 37.1% (95% CI, 17.8–56.6) in those with normal copy number (log-rank test *p* = 0.041) (Figure 2). *MDM4* amplification was not correlated with tumor grade, because the same distribution was observed between amplified and diploid cases. In particular, 5 (25%) of the 20 patients with *MDM4* amplification and 8 (36%) of the 22 patients with no *MDM4* amplification had high-grade tumors.

We considered 5 variables in the Cox regression analysis: age, stage, grade, *MDM4*. In the univariate model, age and *MDM4* CNV were found to be predictive of RFS (Table 2). In particular, patients under 70 years of age showed a greater risk of recurrence than older ones (raw hazard ratio = 3.023) (95% CI, 1.301–7.026). This result was confirmed in multivariate analysis, revealing an adjusted hazard ratio of 3.318 (1.420–7.753; *p* = 0.006). In univariate analysis, patients with a normal *MDM4* copy number had a higher risk of recurrence compared to those with amplified *MDM4* (raw hazard ratio = 2.447) (95% CI, 1.000–5.987; *p* = 0.05). This risk was also confirmed in multivariate analysis (adjusted hazard ratio = 2.745) (95% CI, 1.114–6.766; *p* = 0.028) (Table 2).

Only 4 patients had disease progression, one of which showed amplified *MDM4*.

**Table 2:** Univariate and multivariate analyses for recurrence free survival.

Variables	Categories	Raw HR (95% CI)	*p*	Adjusted HR (95% CI)	*p*
Age, years	<70 *vs.* ≥70	3.023 (1.301–7.026)	0.010	3.318 (1.420–7.753)	0.006
Stage	T1 *vs.* Ta	1.472 (0.605–3.580)	0.394	–	–
Grade	High *vs.* Low	1.369 (0.573–3.269)	0.479	–	–
*MDM4*	N *vs.* G	2.447 (1.000–5.987)	0.05	2.745 (1.114–6.766)	0.028

### 2.2. Discussion

We evaluated a panel of 24 chromosomal aberrations in paraffin-embedded primary NMIBC tissue for their potential to identify patients at high risk of recurrence. An MLPA technique was used that was capable of simultaneously analyzing the entire panel of 24 oncogene copy number variations. A statistically significant association was observed between *MDM4* amplification and a lower risk of NMIBC recurrence.

The *MDM4* gene, located on 1q32.1, encodes a nuclear protein that contains a p53-binding domain which shows a structural similarity to the same domain in *MDM2*. *MDM4* protein acts as an oncogene, inhibiting p53 activity by binding its transcriptional activation domain [16,17]. p53 has been shown to play an important role in bladder cancer tumorigenesis and numerous studies have demonstrated that the gene is often mutated [18] and its expression frequently altered, possibly as a result of altered *MDM2* or *MDM4* genes or proteins [19,20].

In their study of patients with high-grade and -stage bladder cancer, Veerakumarasivam and coworkers observed that *MDM4* gene amplification was mutually exclusive of *p53* mutation. In our case series, *MDM4* gain was seen in 65% of non recurrent NMIBC and in 30% of recurrent cases. It can thus be hypothesized that the p53 pathway is regulated in a different way in the two categories, *i.e.*, via *MDM4* amplification in non recurrent patients and via *p53* mutation in recurrent cases. This is in agreement with the results from a study conducted by George *et al.* in 2007 on a series of mainly invasive bladder cancers [21] in which a positive correlation was identified between mutated *p53* and a high risk of recurrence in patients with bladder cancer. A more comprehensive study of *MDM4* amplification and *p53* mutations and their correlation with disease recurrence in NMIBC is needed to confirm our hypothesis.

The primary objective of our study was to verify any association between copy number variations of selected genes and the risk of local recurrence in NMIBC. Of the 24 genes analyzed, the only statistically significant association was found with *MDM4*. Some other important genes (*MET*, *MYC*, *TOP2A*, *AURKA*, *ERBB2*, *MDM2*, *CCND1*, *BRAF*) were frequently amplified in the overall case series, in agreement with the literature data available on gene copy number alterations in bladder cancer [10,11,12,13,14]. These amplified oncogenes were not correlated with stage or grade but could represent putative therapeutic targets for NMIBC. Indeed, some of the genes, e.g., *ERBB2* and *MET*, are already used as targets for known drugs [22,23].

Bladder cancer is a genetically heterogeneous disease [24] and it would be useful to further our knowledge about gene expression and copy number variations so that new therapeutic targets can be identified and used to tailor treatment.

The present study has some limitations in that it recruited a small number of patients and only used one technique (MLPA) to evaluate copy number variations. The results obtained must now be confirmed in a larger case series and perhaps using a second technique, e.g., real time PCR. A prospective study will be performed to validate our findings.

## 3. Experimental Section

### 3.1. Case Series (Retrospective Cohort Study)

Tissue samples from 43 patients (39 males, 4 females) who underwent transurethral resection of primary bladder cancer in the Department of Urology of Morgagni-Pierantoni Hospital in Forlì between 1997 and 2006 were used for the study. All samples were retrieved from the archives of the Pathology Unit of the same hospital. All patients were in agreement with our biobank and gave written informed consent for their biological material to be used for research purposes (protocol number: 2192/2013, approved by IRST Ethics Committee on 9 May 2013). Median patient age was 72 years (range 39–89): 18 were <70 years and 25 ≥70 years. The final diagnosis, based on 2004 World Health Organization criteria, was low grade non muscle invasive bladder cancer (NMIBC) in 28 patients and high grade NMIBC in 13 patients. At a median follow up of 5 years, 20 patients were still disease-free and 23 had experienced one or more episodes of local recurrence. Patient characteristics are summarized in Table 3.

**Table 3:** Case series.

Clinical and Pathological Characteristics	Patients (*n* = 43)	
	Non Relapsed	Relapsed
Sex		
Male	18	21
Female	2	2
Age, years		
<70	4	14
≥70	16	9
Grade		
High	5	8
Low	14	14
Stage		
Ta	16	16
T1	4	7
Number of tumors		
Single	14	13
Multiple	7	9

### 3.2. Macrodissection and DNA Isolation

Five-micrometer-thick sections were obtained from each paraffin-embedded block and evaluated by hematoxylin-eosin staining. DNA was then isolated from macrodissected cancer tissue only. Genomic DNA was purified using QIAmp DNA FFPE Tissue (Qiagen, Milan, Italy), according to the manufacturer’s instructions. DNA was also isolated from peripheral blood samples from 4 healthy donors using Qiamp DNA Mini Kit (Qiagen, Milan, Italy), according to the manufacturer’s instructions, to be used as a control in further analysis.

### 3.3. Multiplex Ligation Probe Amplification (MLPA) Analysis

MLPA was performed using at least 50 ng of genomic DNA dissolved in 1× TE buffer (Promega, Madison, WI, USA). The amplification status of 24 oncogenes (*MDM4*, *MYCN*, *ALK*, *PDGFRA*, *KIT*, *KDR*, *DHFR*, *EGFR*, *MET*, *SMO*, *FGFR1*, *MYC*, *ABL1*, *RET*, *CCND1*, *CCND2*, *CDK4*, *MDM2*, *AURKB*, *ERBB2*, *TOP2A*, *AURKA*, *AR*, *BRAF*) and *BRAF* V600E mutation were analyzed using P175-A1 kit (MRC-Holland, Amsterdam, The Netherlands) (Table 4). Two different probes that recognize two different sites were used for all but the *CCND2* gene, for which three probes were used.

In brief, DNA was denatured (10 min at 98 °C) and cooled at 25 °C, after which the probe mix was added to the samples and hybridization was performed by incubating at 60 °C for 16–18 h.

A mix composed of ligase-65 buffer, ligase-65 enzyme and water was added and samples were then incubated at 54 °C for 15 min. At the end of the ligation reaction, samples were amplified by adding a mix of PCR buffer, dNTPs and Taq polymerase. The PCR reaction was performed under the following conditions: 37 cycles at 95 °C for 30 s, 60 °C for 30 s and 72 °C for 60 s. The final incubation was performed at 73 °C for 20 min.

**Table 4:** Summary of gene function and chromosomal localization.

Gene	Function	Chromosomal Localization	Exons
*MDM4*	Cell cycle and apoptosis	1q32.1	2,8
*MYCN*	Transcription factor	2p.24	3
*ALK*	Receptor tyrosine kinase	2p23.2	4,6
*PDGFRA*	Cell proliferation and survival	4q12	3,22
*KIT*	Cell proliferation and survival	4q12	2,20
*KDR*	Regulation of angiogenesis	4q12	14,19
*DHFR*	Cell growth and proliferation	5q14.1	1,2
*EGFR*	Cell proliferation	7P11.2	8,22
*MET*	Tumor growth, angiogenesis and metastasis	7q31	4,10
*SMO*	Maintenance of tissue homeostasis	7q32.1	4,12
*FGFR*	Cell proliferation	8p11.2	8,14
*MYC*	Regulation of gene transcription	8q24.1	3
*ABL*	Cell growth and survival	9q34.1	1,12
*RET*	Cell proliferation, cell migration and cell differentiation	10q11.2	8,14
*CCND1*	Cell-cycle regulation	11q13	2,3,5
*TOP2A*	DNA replication, transcription and repair	17q21.2	7,33
*CCND2*	Cell-cycle regulation	12p13.3	1,5
*CDK4*	Cell-cycle regulation	12q14	3,8
*MDM2*	Cell cycle and apoptosis	12q14.3	4,5
*ERBB2*	Transcriptional regulation	17q12	7,29
*AURKB*	Regulator of mitosis	17p13.1	4,5
*AURKA*	Regulator of mitosis	20q13.2	8,10
*AR*	Androgen receptor	Xq12	9

**Figure 3:**
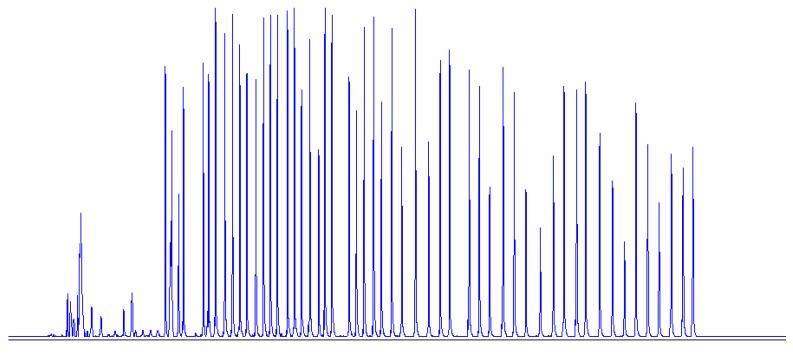
Electropherogram of a control sample (*x*-axis: fragments size (nt); *y*-axis: dye signal).

Amplification products were analyzed by ABI-3130 genetic Analyzer (Applied Biosystems, Cambridge, UK) and 4 reference blood samples were used as controls in each experiment. Electropherograms (Figure 3) were analyzed using Gene Mapper software (Applied Biosystems) and the peak areas of each probe were exported to a self-made spreadsheet. The analysis was performed using Coffalyser software [25], in accordance with the manufacturer’s instructions, and a ratio for each probe was obtained. The final result for each gene was considered as the average of the different probe ratios, which were divided into three categories: “normal” (results between 0.7 and 1.3), “gain” (results > 1.3) and “deletion” (results < 0.7). The *AR* gene was excluded from the analysis because it was not evaluable in paraffin-embedded tissues.

### 3.4. Statistical Analysis

Fisher’s exact test was used to compare the frequency of gene gain in the two subgroups (non recurrent tumors *vs.* recurrent tumors). Due to explorative intent of the study, no corrections were made for multiple testing. RFS was defined as the time between the first diagnosis of primary bladder cancer and the date of the first recurrence. Patients who were still disease-free at database closure were censored at the last follow up. All patients were followed up for at least 5 years.

Cumulative RFS probabilities were estimated using the Kaplan–Meier method and copy number variations of *MDM4* gene were compared using the log-rank test. A Cox regression model was used to estimate RFS hazard ratios (HR) and 95% confidence intervals (CI). The multivariate Cox model included factors that proved significant in the univariate model. All *p*-values were two-sided and a *p* < 0.05 was considered statistically significant. Statistical analyses were performed using SAS 9.3 software (SAS Institute, Cary, NC, USA).

## 4. Conclusions

The present study identified a statistically significant association between *MDM4* amplification and a reduced risk of recurrence of NMIBC. We also observed the frequent amplification of other important genes such as *MET* and *ERBB2*, which could, in future, represent important targets for known drugs. Our findings are key to enhancing the use of personalized medicine in bladder cancer and now require confirmation in larger, prospective studies.
